# Diagnostic Performance of FDG-PET/CT Scan as Compared to US-Guided FNA in Prediction of Axillary Lymph Node Involvement in Breast Cancer Patients

**DOI:** 10.3389/fonc.2021.740336

**Published:** 2021-10-01

**Authors:** Hazem I. Assi, Ibrahim A. Alameh, Jessica Khoury, Maroun Bou Zerdan, Vanessa Akiki, Maya Charafeddine, Ghida I. El Saheb, Fares Sukhon, Eman Sbaity, Serine Baydoun, Nina Shabb, Ghina Berjawi, Mohamad B. Haidar

**Affiliations:** ^1^ Department of Internal Medicine, Division of Hematology and Oncology, Naef K. Basile Cancer Institute, American University of Beirut Medical Center, Beirut, Lebanon; ^2^ Department of Internal Medicine, Division of Endocrinology, American University of Beirut Medical Center, Beirut, Lebanon; ^3^ Department of Radiology, American University of Beirut Medical Center, Beirut, Lebanon; ^4^ Department of General Surgery, American University of Beirut Medical Center, Beirut, Lebanon; ^5^ Department of Pathology, American University of Beirut Medical Center, Beirut, Lebanon

**Keywords:** ^18^F-FDG PET/CT, axillary lymph node, breast cancer, ultrasonography, FNA (fine needle aspiration)

## Abstract

**Purpose:**

The aim of this study was to evaluate the diagnostic ability of 2-deoxy-2-[fluorine-18]fluoro-d-glucose (^18^F-FDG) PET/non-contrast CT compared with those of ultrasound (US)-guided fine needle aspiration (FNA) for axillary lymph node (ALN) staging in breast cancer patients.

**Patients and Methods:**

Preoperative ^18^F-FDG PET/non-contrast CT was performed in 268 women with breast cancer, as well as ALN dissection or sentinel lymph node (SLN) biopsy. One hundred sixty-four patients underwent US-guided FNA in combination with ^18^F-FDG PET/CT. The diagnostic performance of each modality was evaluated using histopathologic assessments as the reference standard. The receiver operating characteristic (ROC) curves were compared to evaluate the diagnostic ability of several imaging modalities.

**Results:**

Axillary ^18^F-FDG uptake was positive in 180 patients, and 125 patients had axillary metastases according to the final pathology obtained by ALN dissection and/or SLN dissection. Of the patients with positive ^18^F-FDG uptake in the axilla, 21% had false-positive results, whereas 79% were truly positive. Eighty-eight patients had negative ^18^F-FDG uptake in the axilla, among which 25% were false-negative. ^18^F-FDG-PET/CT had a sensitivity of 86.59% and a specificity of 63.46% in the assessment of ALN metastasis; on the other hand, US-guided FNA had a sensitivity of 91.67% and a specificity of 87.50%. The mean primary cancer size (*p* = 0.04) and tumor grade (*p* = 0.04) in combination were the only factors associated with the accuracy of ^18^F-FDG PET/CT for detecting metastatic ALNs.

**Conclusion:**

The diagnostic performance of ^18^F-FDG PET/CT for the detection of axillary node metastasis in breast cancer patients was not significantly different from that of US-guided FNA. Combining ^18^F-FDG PET/CT with US-guided FNA or SLN biopsy could improve the diagnostic performance compared to ^18^F-FDG PET/CT alone.

## Introduction

Breast cancer is the most common diagnosed cancer and one of the major causes of cancer-related deaths in female patients worldwide (1). Thus, early detection and accurate evaluation of the extent of the disease spread are an ultimate need. Moreover, preoperative evaluation of the axillary lymph node (ALN) status is crucial for reasons such as estimating prognosis and/or deciding on the suitable treatment plan: whether surgery, chemotherapy, or radiation therapy. Axillary lymph node dissection (ALND), previously used as the primary method for detecting lymph node involvement, is considered invasive and is associated with various life-long complications, some of which are lymphedema, seroma, and upper limb movement restrictions (2, 3).

Sentinel lymph node biopsy (SLNB) is currently performed for eligible breast cancer patients with no evidence of clinical or radiological nodal enlargement. The sentinel lymph node (SLN) is the first node that receives lymphatic drainage from the breast tumor. If the SLN is free of metastasis, then the following lymph nodes are expected to have a negative result as well. As a result, SLNB has replaced ALND and is now the standard procedure to stage patients with clinically node-negative early breast cancer. Despite the advantages of SLNB over ALND, this surgical method remains invasive, time-consuming, and has potential complications (4). Consequently, the application of new accurate and noninvasive imaging modalities to preoperatively assess the axillary status is gradually increasing (5). Such imaging modalities include ultrasound (US)-guided fine-needle aspiration (FNA) and positron emission tomography/computed tomography (PET/CT) scan, both of which have more importance in dictating further therapeutic measures.

Preoperative imaging of the axilla with US is the most used noninvasive management approach for the evaluation of regional lymph nodes. US imaging is considered as the standard of reference for noninvasive imaging techniques in the detection of ALN involvement. It can detect changes in the normal morphology of lymph nodes that suggest metastatic disease. The combination of US and fine-needle aspiration biopsy (FNAB) is a highly accurate non-morbid method for ALN staging (6). The study by Oz. et al. has shown that US-guided FNAB has a sensitivity of 76.6% and a specificity of 100% in assessing ALN involvement in cancer patients. When the US-guided FNAB is positive, SLN dissection can be omitted and patients can directly undergo ALND to complete staging and local control (5).

Another alternative to the invasive techniques is ^18^F-fluorodeoxyglucose positron emission tomography fused with CT (^18^F-FDG-PET/CT). This modality allows detecting increased glucose metabolism, a feature typical of cancerous cells. It is a widely used imaging technique for initial staging, treatment monitoring, and for detecting distant metastasis (7, 8). Riegger et al. showed that intravenous contrast-enhanced ^18^F-FDG-PET may be more accurate than US for the detection of ALN metastases; however, due to its low sensitivity, ^18^F-FDG-PET/CT scan cannot replace SLNB (9). On the other hand, Kim et al. showed that ^18^F-FDG-PET/CT imaging is a specific imaging modality for predicting ALN metastasis, which in turn is helpful in the selective approach to surgical lymph node dissection (10).

Sohn et al. concluded that combining US-guided FNAB and ^18^F-FDG-PET/CT resulted in a significantly higher sensitivity but a lower specificity when compared to FNA or ^18^F-FDG-PET/CT scan alone (3). Nevertheless, a few studies have addressed the diagnostic accuracy of whole-body ^18^F-FDG-PET/CT for ALN staging in comparison to US-guided FNA with ^18^F-FDG-PET/CT.

To the best of our knowledge, there has been no report regarding the combination of US-guided FNA and ^18^F-FDG-PET/CT scan for the preoperative evaluation of the ALN status in breast cancer patients in the Middle East region. Based on all these observations, we aimed to obtain the additional diagnostic performance of US-guided FNA compared with that of an ^18^F-FDG-PET/CT scan to determine the preoperative ALN status in newly diagnosed breast cancer patients. Subsequently, we would like to demonstrate that US-guided FNA could be omitted in cases where ^18^F-FDG-PET/CT is positive. This would reduce not only the number of invasive procedures but also the financial, painful, and psychological burdens on patients.

## Materials and Methods

### Subjects

**Figure 1:**
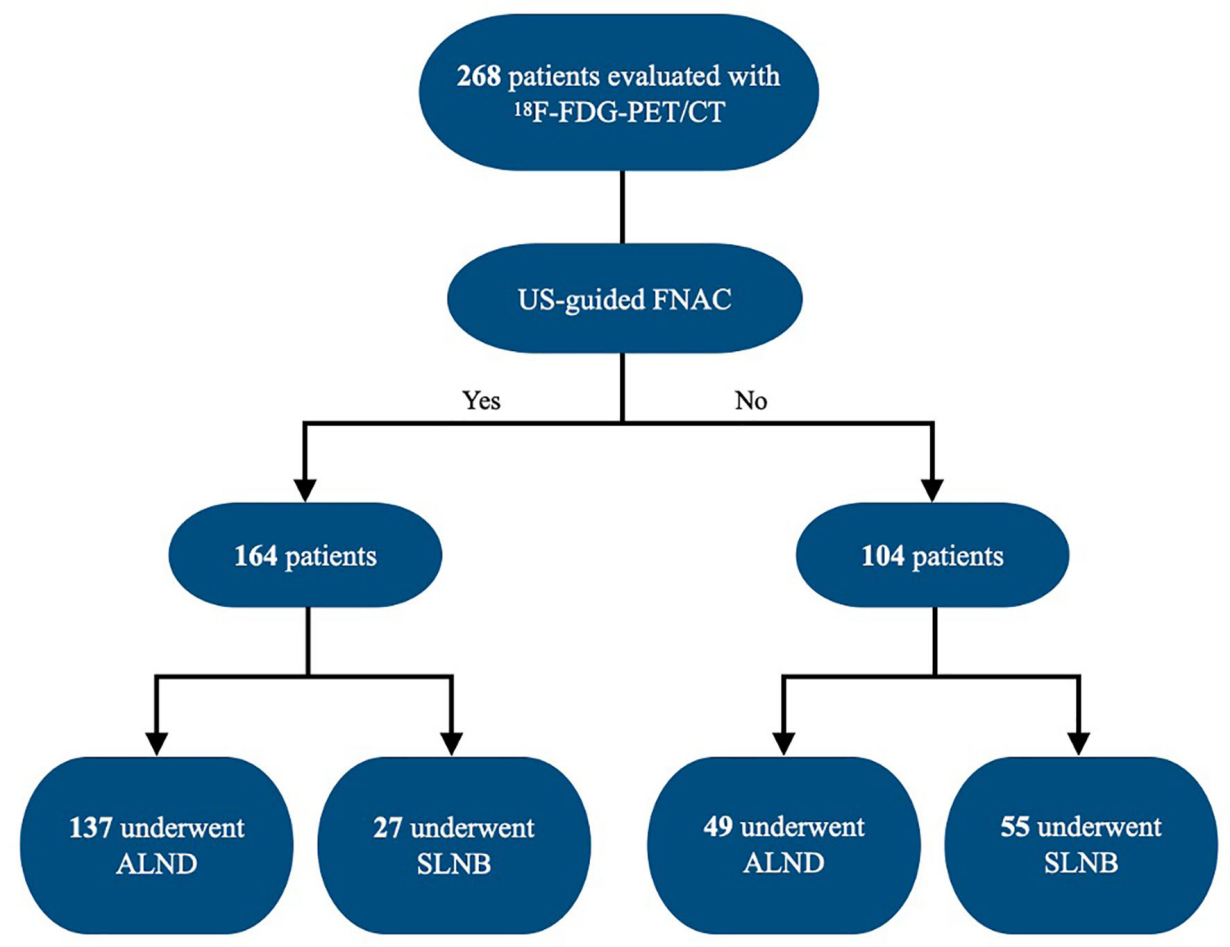
Flowchart of the study group. FNA, fine-needle aspiration; ALND, axillary lymph node dissection; SLNB, sentinel lymph node biopsy.

This was a retrospective chart review-based study that included all women who presented to the American University of Beirut Medical Center between January 1, 2014, and December 31, 2018, with newly diagnosed breast cancer and had invasive ductal carcinoma on excisional or core needle biopsy. Two hundred and sixty-eight patients were evaluated with whole-body ^18^F-FDG-PET/CT and underwent SLNB and/or ALND. Patients with recurrent breast cancer and patients with ductal carcinoma *in situ* were excluded. US-guided fine-needle aspiration cytology (FNAC) was done in 164 patients based on the decision of the primary physician (**Figure 1**).

### Image Acquisition

For the time-of-flight (TOF) FDG-PET imaging protocol, patients were required to fast for at least 6 h prior to scanning. Before intravenous injection of ^18^F-FDG, the blood glucose levels were measured to ensure a value below 15 mmol/L. Patients received an intravenous injection of 180–296 MBq of FDG in the arm contralateral to the primary tumor. At 50–60 min after the injection of FDG, whole-body emission scans were obtained using the Philips GEMINI TF machine (Philips Medical Systems, Cleveland, OH, USA) with the possibility to reconstruct data in 2-mm voxels instead of 5 mm. Patients were in a prone or supine position, with their arms raised. The PET scans were performed using a whole-body PET/CT acquisition protocol with 50% bed overlap. The acquisition time for each patient was 1 min per bed position, with a total of 18 beds. PET data were reconstructed using a default 3D ordered-subset iterative TOF reconstruction technique. The images were reconstructed in two types of matrices: 144 × 144 matrices with a voxel size of 4 × 4 × 4 mm (standard-voxel reconstruction) and 288 × 288 matrices with a voxel size of 2 × 2 × 2 mm (small-voxel reconstruction). Quantitative measurements of the maximal standardized uptake value (SUV_max_) were performed for breast and axillary lesions, when identified. Reconstruction of images in 2 mm centered to the breast and axillary regions was performed for all patients and compared to standard reconstruction.

### Image Analysis

FDG-PET images were evaluated by two experienced nuclear radiologists specializing in PET/CT imaging. Lymph nodes with ^18^F-FDG uptake exceeding that of the surrounding soft tissues were reported as positive. In other words, any focal uptake showing a strong target-to-background ratio compared to the surrounding tissues was considered positive, and any focal uptake with SUV_max_ greater than 2 and did not correspond to physiologic tracer accumulation was considered positive.

Axillary ultrasounds were performed and read by two radiologists. US was performed using a 5.5- to 18-MHz 18L6 HD linear transducer from the Siemens ACUSON S2000 ultrasound system (Siemens, Malvern, PA, USA). Transverse and longitudinal scans were taken and the diameter and cortical thickness of the lymph node(s) were measured. Suspicious lymph nodes were defined based on their shape, border, and echogenicity. Images were interpreted using a dedicated commercially available software, IntelliSpace Portal 8.0, by Philips Healthcare (Amsterdam, Netherlands). This software allows reviewing PET, CT, and fused imaging data in the axial, coronal, and sagittal planes. US-guided FNAC was performed on suspicious lymph nodes. A 20-ml syringe and a 3.8-cm-long 20-gauge needle or a 5-cm-long 21-gauge needle was used. The needle was inserted at a very shallow angle in order to remain as close to parallel to the pleura as possible for maximum safety, and the aspirate of cellular material was sent to the pathology laboratory for examination. Given the retrospective nature of the study, the decision to obtain an US-guided FNA was left to the primary physician. FDG-avid ALNs from the ^18^F-FDG-PET/CT scan were correlated with the positive lymph nodes on the US-guided FNAC by our radiologists.

### Statistical Analysis

The overall accuracy was calculated as the percentage of all true positives and true negatives compared to the total number of cases. The pathology of ALND or SLNB was considered as the reference standard. Univariate and multivariate logistic regression analyses were conducted to identify the factors affecting the results of ^18^F-FDG-PET/CT for axillary metastasis. All data were analyzed using SPSS v.23.

### Ethics Committee Approval and Consent

This retrospective study was reviewed and approved by the independent Ethical Committee of the American University of Beirut Medical Center (IM.HA.13). All procedures involving human participants were consistent with the ethical standards of the institutional and/or national research committee and with the 1964 Helsinki Declaration and its later amendments or comparable ethical standards. The need for informed consent was waived. The demographic data and clinical characteristics of the patients, such as age at diagnosis, histologic type of breast cancer, stage, BMI, and smoking status, were collected from the medical charts. Moreover, morphometric variables such as the number of detected suspicious lymph nodes, PET uptake, and SUV_max_ were obtained. The sensitivity, specificity, false-negative rate (FNR), and the false-positive rate (FPR) were calculated for ^18^F-FDG-PET/CT and for US-guided FNAC.

## Results

A total of 268 patients were included in this study. The patient demographics and clinical characteristics are listed in **Table 1**. The mean age of the patients was 49.74 years, with a range between 23 and 84 years. The mean size of the primary breast cancer obtained by surgery was 2.1 ± 2.3 cm. Most patients had T2 stage breast cancer (41.8%), followed by T1 stage (32.9%). The majority had estrogen receptor (ER)-positive (77.6%) and progesterone receptor (PR)-positive breast cancer (67.2%), and only 34.3% of patients had positive HER2 receptors. Regarding the histologic grade, 129 patients had high-grade tumors (grade 3), while only 25 had tumors with histologic grade 1.

**Table 1:** Demographics and tumor characteristics.

Characteristic (*N* = 268)	Value	Percentage
Mean age (years) (range)	49.74 (23–84)	
Mean primary cancer size ( ± SD)	2.1 ( ± 2.3)	
T stage^a^		
T1	74	32.9
T2	94	41.8
T3	30	13.3
T4	27	12.0
N stage		
pN0	73	33.1
pN1	103	46.6
pN2	27	12.2
pN3	18	8.1
ER		
Positive	208	77.6
Negative	60	22.4
PR		
Positive	180	67.2
Negative	88	32.8
HER2		
Positive	92	34.3
Negative	176	65.7
Histologic grade		
Grade 1	25	9.5
Grade 2	108	41.3
Grade 3	129	49.2

^a^Categorized by the American Joint Committee on Cancer.

Out of the 268 patients who underwent ^18^F-FDG-PET/CT, 164 patients underwent both ^18^F-FDG-PET/CT and US-guided FNAC. One hundred twenty-eight patients had positive lymph nodes on the cytology results and 39 patients had negative results. One hundred seventeen patients had concordant results on ^18^F-FDG-PET/CT and US-guided FNAC (both modalities were positive for lymph node involvement or both were negative); 30 patients had discordant results.

**Figure 2:**
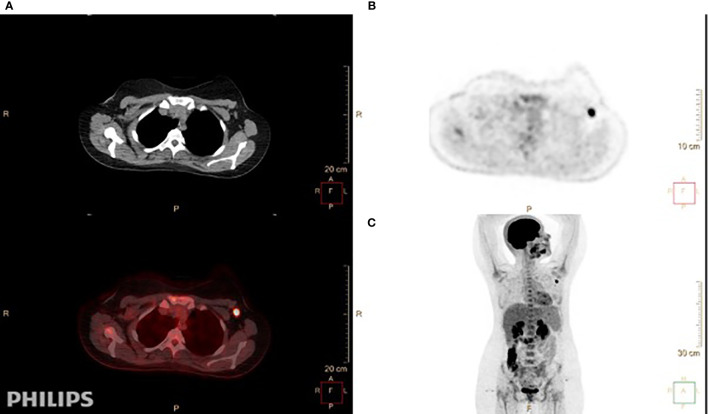
A 24-year-old-female patient with invasive ductal carcinoma in the left breast, grade II, estrogen receptor (ER)-positive, progesterone receptor (PR)-positive, and HER2-negative. She had T1, N1, Mx disease. **(A)** Axial ^18^F-FDG-PET/CT showed fluorodeoxyglucose (FDG)-avid left axillary lymph nodes. The largest and most avid measures 1 × 1.6 cm with SUV_max_ of 9.8. **(B, C)** Axial **(B)** and coronal **(C)** views demonstrate high FDG uptake for the enlarged node in the left axilla.

As seen in **Table 2**, which describes the tumor and lymph node characteristics determined by ^18^F-FDG-PET/CT, the SUV_max_ of the ALN was 5.88 and ranged between 0.6 and 30, while the SUV_max_ of the primary tumor was 7.44, ranging from 1.1 to 43. The mean size of the ALNs by ^18^F-FDG-PET/CT was 1.56 cm and that of the tumor was 2.59 cm (**Figure 2**).

**Table 2:** Tumor and lymph node characteristics by PET/CT (*N* = 268).

	Mean ± *SD*	Min–Max	Median
Tumor size determined by ^18^F-FDG-PET/CT (cm)	2.59 *± 2.1*	0.6–20.2	2.2
Tumor SUV_max_ determined by ^18^F-FDG-PET/CT	7.44 *± 5.91*	1.1–43	5.75
ALN size determined by ^18^F-FDG-PET/CT (cm)	1.56 *± 1.3*	0.3–7	1.3
ALN SUV_max_ determined by ^18^F-FDG-PET/CT	5.88 *± 5.9*	0.6–30	3.8

SUV_max_, maximal standardized uptake value; ALN, axillary lymph node.

Axillary ^18^F-FDG uptake was positive in 180 patients and negative in 88 patients (**Table 3**). On the other hand, 164 patients had axillary involvement according to the final pathology obtained by ALND and/or SLNB, while the remaining 104 patients had no axillary metastases. In addition, ^18^F-FDG-PET detected extra-axillary FDG-avid lymph nodes (internal mammary, supraclavicular, and mediastinal) in 19 patients.

**Table 3:** Relationship between the ^18^F-FDG-PET/CT findings and the histological involvement of axillary lymph nodes following surgery.

	No. of cases	Histological involvement of ALN following surgery		Total
		Negative	Positive	
Findings of 18F-FDG-PET/CT	Negative	66	22 (25%)	88
	Positive	38 (21%)	142	180
	Total	104	164	268

ALN, axillary lymph node.

Thirty-eight patients out of 180 with positive ^18^F-FDG uptake in the axilla (21%) had a false-positive result, whereas 22 (25%) had a false-negative result (**Table 3**).

It has to be noted that, upon further subset analysis of the patients who had US-guided FNA before undergoing ^18^F-FDG-PET/CT (*N* = 87), six of these patients had a false-positive result. Thus, these six patients out of the 38 with a false-positive result mentioned in **Table 3** might have had a false result because of an inflammatory reaction following the FNA.

The diagnostic performance of each modality is shown in **Table 4**. ^18^F-FDG-PET/CT had a sensitivity of 86.59% and a specificity of 63.46% in the assessment of ALN metastasis; on the other hand, US-guided FNAC had a sensitivity of 91.67% and a specificity of 87.5%. When combining the two modalities, ^18^F-FDG-PET/CT and US-guided FNAC, we obtained a sensitivity of 62.5% and a specificity of 90.85%.

**Table 4:** Diagnostic performance of PET-CT and FNA in the assessment of lymph node metastasis.

Type of imaging	Sensitivity (%)	Specificity (%)	PPV (%)	NPV (%)	Accuracy (%)
^18^F-FDG-PET/CT	86.59	63.46	78.89	75.00	77.61
US-guided FNA	91.67	87.50	96.80	71.79	90.85
Combination of ^18^F-FDG-PET/CT with US-guided FNA	90.85	62.50	79.26	81.25	79.85

PPV, positive predictive value; NPV, negative predictive value; US, ultrasound; FNA, fine-needle aspiration.

**Figure 3:**
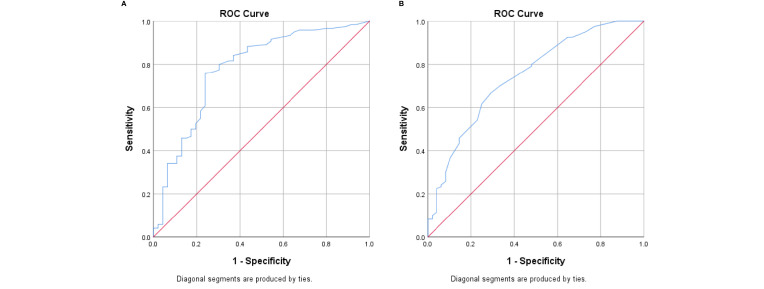
**(A)** Receiver operating characteristic (ROC) curve analysis of the SUV_max_ of lymph nodes. **(B)** ROC curve analysis of lymph node size.

In addition, the SUV_max_ values of metastatic lymph nodes were significantly higher than those of benign lymph nodes (*p* < 0.001). According to the receiver operating characteristic (ROC) curve analysis (**Figures 3A, B**), the diagnostic performance was significantly better when the cutoff value of SUV_max_ was 2.55 and the cutoff size of the lymph nodes was 1.05 cm.

As seen in **Table 5**, the higher the T stage, the higher the SUV_max_ values of the tumor (*p* < 0.001) and that of the lymph nodes (*p* < 0.001).

**Table 5:** Relationship between T stage and the mean SUV_max_ of tumor and ALN (*N* = 268).

T stage	Mean SUV_max_ of the tumor (*p* < 0.001)	Mean SUV_max_ of ALNs (*p* < 0.001)
1	4.58	4.13
2	8.19	5.59
3	7.04	5.84
4	11.65	9.55

SUV_max_, maximal standardized uptake value; ALN, axillary lymph node.

A univariate analysis was performed to evaluate the relationship between the true-negative and the false-negative results of the ALNs between ^18^F-FDG-PET/CT and the final pathology results (**Table 6**).

**Table 6:** Univariate analysis of the factors affecting the true-negative and false-negative results of ^18^F-FDG-PET/CT for axillary metastasis (*N* = 268).

Characteristic	True negative	False negative	*p*-value
Histologic grade			0.094
Grade 1	14	1	
Grade 2	24	12	
Grade 3	27	9	
ER			0.685
Negative	6	3	
Positive	66	22	
PR			0.399
Negative	13	4	
Positive	53	18	
HER-2			0.574
Negative	46	16	
Positive	20	6	

ER, estrogen receptor; PR, progesterone receptor.

On multivariate analysis, the mean primary cancer size (*p* = 0.04) and the tumor grade (*p* = 0.04) in combination were the only factors associated with the accuracy ^18^F-FDG-PET/CT for detecting metastatic ALNs. In fact, the higher the mean primary cancer size and the higher the grade of the tumor, the less likely that ^18^F-FDG-PET/CT will miss the ALNs.

## Discussion

Various imaging modalities, such as US, MRI, and ^18^F-FDG-PET/CT, have played a key role in breast cancer staging and management. ^18^F-FDG-PET/CT has the advantage of allowing examination of the whole body, including the bones, chest, and abdominal organs, in one session (11). The ALN status is a main factor in breast cancer prognosis since preoperative staging of ALNs is essential for surgery guidance and the selection of aggressive local therapy (1). In this study, we retrospectively compared the ability of ^18^F-FDG-PET/CT against US-guided FNA in detecting lymph node metastases.

The sensitivity and specificity of ^18^F-FDG-PET/CT in detecting ALNs have varied among many studies, with a wide range of sensitivities between 20% and 100% and specificities between 64% and 97% (12–20). In our study, ^18^F-FDG-PET/CT had a sensitivity of 86.59% and a specificity of 63.46% in the assessment of ALN metastasis, while US-guided FNAC had a sensitivity of 91.67% and a specificity of 87.50%. Both had comparable sensitivity values, which could open a new pathway for preoperative planning of patients. This would also be beneficial in terms of the financial burden and physical pain from invasive procedures. However, false-negative results can still occur. In our study, 25% of patients had false-negative results, which is similar to that in the study by Nakano et al. showing a false-negative result rate of 22% (4).

Furthermore, the combination of both modalities maintained a sensitivity of 90.85%, but a fall in specificity was marked compared to US-guided FNAC alone (62.50% *vs*. 87.50%). Compared to the results of Nakano et al., our cohort showed higher sensitivity and accuracy values when combining ^18^F-FDG-PET/CT and US-guided FNAC (4). With the positive predictive value (PPV) reaching 79.26%, patients could initially undergo ^18^F-FDG-PET/CT to evaluate regional and distant metastases. A randomized clinical trial is needed to confirm our findings prior to the implementation of new guidelines based on skipping US-guided FNAC as the gold standard in patients with a positive FDG uptake above a certain SUV_max_ cutoff. On the other hand, going for an US-guided FNAC or US sampling for suspicious lymph nodes is an option in some settings. These settings include situations where there is no hypermetabolic activity in regional lymph nodes or when the specificity and the negative predictive value (NPV) of a modality are low. Therefore, ^18^F-FDG-PET/CT could be considered as a pretest for invasive locoregional staging procedures.

As shown in **Figure 3**, the diagnostic performance was significantly better when the cutoff lymph node size was 1.05 cm. In fact, many studies have reported that the inferior sensitivity of ^18^F-FDG-PET/CT was related to micro-metastasis and considered it as an important limiting factor (21). In a study by Yararbas et al., five patients out of 32 had false-negative ALNs on ^18^F-FDG-PET/CT; three had millimetric metastatic foci (1). In our study, the higher the T stage, or the higher the grade, the higher the likelihood of the accurate detection of axillary metastasis by ^18^F-FDG-PET/CT (**Table 6**).

Moreover, the specificity of ^18^F-FDG-PET/CT in the present study was shown to be 63.46%, with a PPV of 78.89%. The finding of an FDG-avid axillary node is expected to represent a malignant lymph node. SLNB can be avoided and the surgeon can directly proceed with ALND whenever a patient has a positive ^18^F-FDG uptake in the axilla. This goes back to the high PPVs of ^18^F-FDG-PET/CT seen in some studies (20, 22, 23). Nevertheless, false-positive results can occur for several reasons, such as inflammation following a procedure in the axillary area and reactive lymphadenopathy following biopsy of the breast (24, 25). In fact, 87 patients in our study had US-guided FNAC before undergoing ^18^F-FDG-PET/CT. Six of them had a false-positive result, which could have been attributed to these reasons and could have lowered the overall specificity. At the same time, the higher the mean primary cancer size and the higher the grade of the tumor, the less likely that ^18^F-FDG-PET/CT will miss the ALNs (**Table 7**). This is further shown in **Table 5**; the higher the T stage, the higher is the SUV_max_ of the lymph nodes (*p* < 0.001). In this present study, the optimal cutoff value of SUV_max_ was 2.55. In the study by Kutlutürkhe et al., when the SUV_max_ of ALNs was higher than 3.2, the likelihood of ^18^F-FDG-PET/CT being accurate for axillary metastasis was 15.6 higher (26). Thus, the optimal value should be taken into consideration along with visual information when relying on PET/CT.

**Table 7:** Multivariate analysis of the factors affecting the true-positive/true-negative *versus* false-positive/false-negative results of axillary metastasis.

	p-value	Coefficient	95% Confidence interval
Mean primary cancer size (cm)	0.04	1.29	1.04–1.60
Age	0.37	1.01	0.98–1.04
ER (negative *vs*. positive)	0.93	0.97	0.28–3.31
PR (negative *vs*. positive)	0.99	0.99	0.37–2.92
HER-2 (negative *vs*. positive)	0.26	1.67	0.73–3.54
Histologic grade			
Grade 1	0.09		
Grade 2	0.05	0.26	0.04–0.97
Grade 3	0.04	0.17	0.03–0.82
T stage			
T1	0.21		
T2	0.16	0.56	0.25–1.22
T3	0.83	0.89	0.26–2.97
T4	0.35	2.01	0.50–8.11

ER, estrogen receptor; PR, progesterone receptor.

In addition, locoregional nodes such as the internal mammary and the infra/supraclavicular nodes can be less likely identified through the SLN technique. Therefore, identifying these extraaxillary lymph node metastases using ^18^F-FDG-PET/CT has an influential value in cancer staging and affecting prognosis. In fact, it can even change the planned therapy (27). The recent 2019 guidelines allow the optional use of ^18^F-FDG-PET/CT for the initial staging of locally advanced diseases and the evaluation of treatment response, stating that “FDG PET/CT may also be helpful in identifying unsuspected regional nodal disease and/or distant metastases” and that “FDG PET/CT is most helpful in situations where standard staging studies are equivocal or suspicious, especially in the setting of locally advanced or metastatic disease” (28).

Efforts to utilize artificial intelligence (AI) have been prominent in recent years. In a recent study by Li et al. (29), more than 400 patients were retrospectively included as part of a comparative study between two clinicians and AI. Four hundred and fourteen axillae from patients with biopsy-proven breast cancer who had also undergone ^18^F-FDG-PET/CT before undergoing SLNB and/or ALND were included in the study. Although the AI model, a designed and trained 3D convolutional neural network, did not overtake the clinicians, the accuracies were improved, with sensitivity values from 59.8% and 57.4% to 68.6% and 64.2%, but the specificities remained unchanged (29).

Lastly, tumor markers have been recently utilized in asymptomatic breast cancer patients receiving adjuvant therapies. These tumor markers have been shown to be significantly predictive of distant metastases identified on FDG-PET/CT. In a study by Corso et al., cancer antigen 15-3 (CA 15-3) and carcinoembryonic antigen (CEA) were analyzed in 561 patients (30). The median value of CA 15-3 was 35.0 U/ml in cases where no metastases were detected and was 58.9 U/ml in cases with positive metastases (*p* < 0.001). Similarly, the CEA values were 6.6 *vs*. 12.4 U/ml (*p* < 0.001). Furthermore, CA 15-3 had a significant association with bone/liver metastases compared to other sites of metastasis (30). This opens up other future viable options as staging procedures.

There were several limitations to this study. Firstly, this study was retrospective in nature, and this could have affected the data represented. For this reason, further prospective studies with larger populations are anticipated, with control of ^18^F-FDG-PET/CT preceding US-guided FNAC to exclude false positivity. Secondly, not all patients underwent US-guided FNAC. Thirdly, most patients had high-risk breast cancers (T2–T4 > T1; N1 > N0). Consequently, early breast cancer with low-risk ALN metastases could not be assessed. Also, the lymph nodes were not marked at the time of ALND or US-guided FNA, which could have ensured that the same node is being studied with each modality.

## Conclusion

In conclusion, ^18^F-FDG-PET/CT has a promising future in staging breast cancer and tailoring the treatment plan. Not only does it help in detecting extra-axillary nodal and metastatic disease, but it also helps in detecting ALNs in a noninvasive way and allows viewing the whole body at a single point of time. However, preoperative ALN staging using ^18^F-FDG-PET/CT as a single modality is not sufficient. Therefore, a combined evaluation (sonography, FNA, ^18^F-FDG-PET/CT, and SLNB) is preferred.

## Data Availability Statement

The raw data supporting the conclusions of this article will be made available by the authors, without undue reservation.

## Ethics Statement

The studies involving human participants were reviewed and approved by the American University of Beirut Medical Center. The patients/participants provided written informed consent to participate in this study.

## Author Contributions

IA, JK, VA, and MBZ wrote the manuscript. MC, HA, IA, VA, SB, GB, and MBZ analyzed and interpreted the data. IA, JK, VA, GES, and NS collected and/or assembled the data. HA, ES, SB, NS, GB, and MH provided study materials or patients. HA, VA, FS, ES, and MH conceived and designed the study. All authors contributed to the article and approved the submitted version.

## Conflict of Interest

The authors declare that the research was conducted in the absence of any commercial or financial relationships that could be construed as a potential conflict of interest.

## Publisher’s Note

All claims expressed in this article are solely those of the authors and do not necessarily represent those of their affiliated organizations, or those of the publisher, the editors and the reviewers. Any product that may be evaluated in this article, or claim that may be made by its manufacturer, is not guaranteed or endorsed by the publisher.
